# Gastrodia elata fermentation alleviates methamphetamine-induced neuroinflammation and anxiety- and depression-like behaviors by regulating the PI3K-AKT signaling pathway

**DOI:** 10.3389/fmed.2026.1768944

**Published:** 2026-01-27

**Authors:** Yubo Liu, Hansen Yang, Hongying Li, Shan Zhang, Han Yang, Yu Luo, Ting Hu, Zhenting Zhang, Xiaobo Peng, Yuanhe Wang, Shaofeng Wei, Bing Xia, Peng Luo

**Affiliations:** 1 Key Laboratory of Environmental Pollution and Disease Monitoring, Ministry of Education, School of Public Health, Guizhou Medical University, Guiyang, China; 2 National Key Laboratory of Exploration and Utilization of Effective Components of Traditional Chinese Medicine, Guiyang, China; 3 College of Forensic Medicine, Guizhou Medical University, Forensic Judicial Appraisal Center, Guiyang, China; 4 The Provincial Department of Guizhou Medical University Jointly Established a Collaborative Innovation Center for Disease Prevention and Control in Endemic and Ethnic Areas, Guiyang, China; 5 The Second Affiliated Hospital of Guizhou Medical University, Kaili, China; 6 Guizhou Industrial Technology Development Research Institute, Guiyang, China

**Keywords:** anxiety, depression, Gastrodia elata fermentation (FGE), methamphetamine, neuroinflammation, PI3K-AKT pathway

## Abstract

**Background:**

Intractable depression- and anxiety-like behaviors significantly contribute to methamphetamine (METH) abuse and relapse, which are linked to METH-induced neuroinflammation and impaired neural function. Fermented Gastrodia elata (FGE) is produced through a specific fermentation process. Previous studies have shown that its primary active components, including gamma-aminobutyric acid (GABA) and 4-hydroxybenzyl alcohol (4-HBA), exhibit notable anti-inflammatory and neuroprotective properties. However, the protective effects of FGE against METH-induced neuroinflammation in the brain and the underlying mechanisms remain incompletely understood.

**Objective:**

This study aims to investigate the potential protective effects of FGE against METH-induced neuroinflammation in hippocampus neurons and its impact on anxiety- and depression-like behaviors in mice. Additionally, we seek to elucidate the underlying molecular mechanisms.

**Methods:**

A mouse model of anxiety- and depression-like behaviors was established using METH induction. Pathological changes in hippocampus neurons were examined via H&E staining. The effects of FGE on these neurons were evaluated through pathological analysis. A series of behavioral tests were conducted to assess the impact of FGE on METH-induced depressive- and anxiety-like behaviors. To further investigate the molecular pathways underlying the neuroprotective effects of FGE, network pharmacology, ELISA, and RT-PCR were employed.

**Results:**

The results demonstrated that METH effectively induced anxiety- and depression-like behaviors, which were associated with hippocampus neuroinflammation and neuropathology, along with significant activation of astrocytes and microglia. Intervention with FGE notably improved hippocampus pathology, reduced glial cell activation, and alleviated the associated anxiety- and depression-like behaviors. Furthermore, network pharmacology analysis suggested that the PI3K-AKT signaling pathway may contribute to the protective effects of FGE against METH-induced neuronal injury.

**Conclusion:**

This study demonstrates that FGE, a potential natural agent, alleviates anxiety- and depression-like behaviors induced by METH through the reduction of neuroinflammation in the hippocampus. The protective effects of FGE against METH-induced neuronal damage and behavioral deficits are likely mediated by the PI3K-AKT signaling pathway. These findings suggest FGE as a promising therapeutic strategy for METH-related neurological disorders.

## 1 Introduction

Methamphetamine (METH), also known as methylamphetamine or desoxyephedrine, is a potent psychostimulant. High-purity METH crystals resemble rock candy, which is why they are often referred to as “ice “. Due to its ease of synthesis, high addictive potential, and severe neurotoxicity, METH abuse has escalated into a critical global public health crisis^1^ (1). According to the 2024 World Drug Report, amphetamine-type drugs were abused in most countries around the world in 2022 (see text footnote 1). Upon administration, METH is distributed to vital organs, including the brain, heart, and kidneys, resulting in significant neurotoxicity both peripherally and centrally (2). Its central neurotoxic effects are characterized by extensive damage to dopaminergic nerve terminals and exacerbated neuroinflammation (3). Damage to the central nervous system induced by METH is pivotal in the etiology of persistent and intractable negative emotional disorders, including anxiety, depression, and anhedonia (4). Therefore, addressing the mood symptoms induced by METH is essential for reducing relapse rates. Currently, there is a significant lack of safe and effective pharmacological interventions to counter METH-mediated neurotoxicity and the related emotional impairments. This highlights the urgent need for the development of novel neuroprotective therapeutics.

Astrocytes and microglia, the two principal glial cell types in the central nervous system (CNS), play critical roles in maintaining homeostasis. Astrocytes are primarily responsible for blood–brain barrier integrity and synaptic homeostasis, while microglia regulate brain development and respond to damage and pathogens (5). When the microenvironment surrounding glial cells is altered, these cells initiate a series of responses to maintain cerebral homeostasis. Activated microglia phagocytose abnormal substances and release pro-inflammatory cytokines, which amplify the inflammatory response and contribute to neuronal damage (6). Glial cell-mediated neuronal damage is associated with the activation of NF-κB, MAPK, and phosphatidylinositol 3-kinase (PI3K)/protein kinase B (AKT) signaling pathways. Activation of these pathways leads to the release of significant amounts of pro-inflammatory factors, thereby increasing their neurotoxic potential (7).

Methamphetamine (METH) exposure can significantly activate astrocytes and microglia. The persistent activation of these cells initiates an inflammatory cascade (8, 9), which promotes neuronal injury through the overproduction of inflammatory factors (10). This process ultimately disrupts neural circuitry in emotion-related brain areas, such as the prefrontal cortex and hippocampus. Consequently, information transmission and integration are impaired, leading to the emergence of emotional deficits (11, 12). Collectively, glial activation driven by METH is a crucial mechanism contributing to neuroinflammation and central functional impairments. Therefore, targeting and suppressing this activation is considered a critical strategy for alleviating METH-induced neurological damage and associated anxiety or depressive behaviors.

Contemporary research on Traditional Chinese Medicine has increasingly focused on the potential of natural products to alleviate emotional disorders associated with methamphetamine use.

Modern research on Traditional Chinese Medicine has increasingly emphasized the potential of natural products in mitigating emotional disorders induced by METH. Notable examples with documented pharmacological efficacy include *Pueraria lobata* (13), rhynchophylline (14), Dictyophora indusiata (15), and, notably, Gastrodia elata Bl. (16). Gastrodia elata Bl. (Orchidaceae) is a traditional medicinal plant whose dried tuber is used. Key bioactive constituents isolated from Gastrodia elata, such as gastrodin and gamma-aminobutyric acid (GABA), have been shown to possess neuroprotective properties, among other biological activities (17, 18). Fermented Gastrodia elata (FGE) is a product derived from the fermentation of the Gastrodia elata Bl. Compared to unfermented Gastrodia elata, FGE demonstrates significant improvements in flavor, functional compound content, and antioxidant capacity (19). Previous studies have shown that FGE can elevate the levels of GABA and superoxide dismutase (SOD) in the hippocampus of animal models of anxiety and depression (20). Furthermore, it has been shown to alleviate depressive-like behaviors in murine models by upregulating the expression of monoamine neurotransmitters and brain-derived neurotrophic factor (BDNF) protein, thereby mitigating neuronal damage (21). These findings indicate that FGE may have the potential to alleviate METH-induced anxiety and depression. However, it remains unclear whether FGE achieves this by modulating METH-induced neuroinflammation, which could in turn reduce anxiety- and depression-like behaviors in mice. To address this question, the present study combined network pharmacology predictions with *in vivo* experimental validation.

This study investigated the effects of FGE on METH-induced neuroinflammation and anxiety- and depressive-like behaviors in mice. We aimed to determine whether these effects are mediated through the regulation of glial cell activation via specific signaling pathways. The research provides experimental evidence supporting FGE as a potential intervention for METH-related neuropsychiatric disorders.

## 2 Materials

### 2.1 Reagents and kits

SP Rabbit/Mouse HRP Kit (DAB) (Cat# SA00001-2/SA00001-1, CWBIO, China); DAB Substrate Kit (Cat# ZLI-9018, Zhongshan Jinqiao, Beijing, China); SPARKscript II RT Kit/SYBR Green RT-PCR Kit (Cat# AG0305-B/AH0104-B, SparkJade, China); Anti-Iba1 Rabbit Polyclonal Antibody (Cat# F155306, Abways, China); Anti-GFAP Rabbit Polyclonal Antibody (Cat# CY5424, Abcam, United Kingdom); Anti-NeuN Rabbit Polyclonal Antibody (Cat# 26975-1-AP, Proteintech, China); AKT/phosphorylated AKT (p-AKT)/phosphorylated PI3K (p-PI3K)/GAPDH (Cat# 10176-2-AP/66444-1-IG/20584-1-AP/60004-1-IG, Proteintech, China); PI3K (Cat# AF3242, Affinity, China).

Allegra X-30R High-Speed Refrigerated Centrifuge (Beckman Coulter, United States); ME-204 Electronic Analytical Balance (Mettler Toledo, Switzerland); Direct-Q^®^ 3 UV Water Purification System (MilliporeSigma, United States); VisuTrack 3.0 Behavioral Analysis Software (Shanghai Softmaze Information Technology Co., Ltd., China); EasyScan Digital Slide Scanner and Application System (Motic, China); 7,500 Fast Real-Time PCR System (Applied Biosystems, Thermo Fisher Scientific, United States); μQuant Microplate Spectrophotometer (BioTek Instruments, Agilent, United States).

### 2.2 Experimental drugs

Methamphetamine (METH), a white crystalline powder with a purity of ≥ 98%, was supplied by the Division of the Guiyang Municipal Public Security Bureau (Guizhou, China). The substance was dissolved in normal saline to achieve the required concentrations and stored at 20 °C.

FGE: An orange-red, clear liquid formulation of FGE was prepared according to the protocols established in our previous studies. The extract was stored under standard conditions and freshly prepared prior to use.

### 2.3 Experimental animals

Male C57BL/6 J mice, aged 8 weeks and weighing 20 ± 2 g, were obtained from the Experimental Animal Center of Guizhou Medical University (Guiyang, China); Animal Use License No. SYXK (Gui, 2025-0001). All mice were specific pathogen-free (SPF) grade. They were housed under standard laboratory conditions, including a constant temperature of 22–26 °C, relative humidity of 50 5%, and a 12/12-h light/dark cycle. Food and water were provided ad libitum. Prior to experimental procedures, the mice were acclimatized to the housing facility for 1 week.

All animal experiments were approved by the Institutional Animal Care and Use Committee (IACUC) of Guizhou Medical University (No. 2500585).

## 3 Experimental methods

### 3.1 Network pharmacology-based screening of medicinal herbs with potential therapeutic effects

The chemical constituents of fermented Gastrodia elata (FGE) were identified through a systematic search of the PubMed database using the keyword “Fermented Gastrodia elata. “After removing duplicates, comprehensive information for each unique compound was retrieved from the Herb database.^2^ To focus on compounds with favorable drug-like properties, we applied Lipinski’s Rule of Five (number of hydrogen bond donors ≤5, number of hydrogen bond acceptors ≤10, LogP ≤5, molecular weight ≤ 500 Da, number of rotatable bonds ≤10) to screen for potential active ingredients. The canonical SMILES notations were subsequently used as input for the Swiss Target Prediction^3^ platform to predict their putative protein targets in *Homo sapiens*. Simultaneously, disease-associated targets related to “anxiety” and “depression” were collected from three major databases: Genecards^4^, OMIM^5^, DrugBank.^6^ All target identifiers from the compound prediction and disease association steps were standardized to official gene symbols and rigorously deduplicated. This process yielded unified target datasets for “anxiety” and “depression. “The intersection of these two disease-specific target sets was then calculated to generate a core anxiety-depression target dataset, representing genes potentially involved in the comorbidity of these conditions. To visualize protein–protein interactions (PPIs), the component target–anxiety–depression target dataset was imported into the STRING database^7^ to generate a PPI network. Subsequently, a network representing “active component targets–anxiety and depression targets” was constructed in Cytoscape. Using the topological analysis tools in Cytoscape, we calculated node degree values and edge counts, leading to the generation of a network diagram titled “Active Components–Anxiety and Depression–Targets.” Finally, we performed Gene Ontology (GO) and Kyoto Encyclopedia of Genes and Genomes (KEGG) pathway enrichment analysis on the core subnetwork related to depression and anxiety targets using the DAVID platform.^8^

### 3.2 Molecular docking

The disease target with the highest degree value was selected as the receptor, while the active ingredient served as the ligand. The protein structure of the receptor was retrieved from the Protein Data Bank (PDB) by searching for the corresponding UniProt entry. This was followed by preprocessing to generate the PDBQT file. The 3D structure of the ligand was obtained in SDF format from PubChem and was energy-minimized using the MM2 force field in ChemBio3D. It was then saved as a MOL2 file and subsequently converted to PDBQT format using Meeko.

To define the center and dimensions of the docking box, a “.dock” file generated with Dockey was parsed, creating the configuration file for Vina. Molecular docking was performed using AutoDock Vina 1.2.5, with uniform grid parameters set to spacing = 0.375 Å, exhaustiveness = 16, num_modes = 20, and energy_range = 4. For each receptor-ligand pair, the conformation with the lowest binding affinity was chosen as the optimal docking pose for further visualization and analysis.

### 3.3 Experimental models

Male C57BL/6 J mice were randomly assigned to six groups (*n* = 6): (1) Control group, (2) FGE group, (3) METH group, (4) METH + low-dose FGE group (METH+FGE-L), (5) METH + medium-dose FGE group (METH+FGE-M), and (6) METH + high-dose FGE group (METH+FGE-H). The experiment comprised two phases: an induction phase and an intervention phase. During the 4-day induction phase, mice in the METH, METH+FGE-L, METH+FGE-M, and METH+FGE-H groups received intraperitoneal (i.p.) injections of METH (15 mg/kg, twice daily). Mice in the Control and FGE groups received equivalent volumes of saline via i.p. injection. Body weight was recorded daily for all animals. Following the induction phase, a 28-day intervention period commenced (In rodent models, partial spontaneous recovery from METH-induced neurotoxicity can be observed approximately 14 days after cessation (22, 23). To ensure that the improvement in anxiety- and depression-like behaviors in mice in this experiment was attributable to the efficacy of FGE intervention and to achieve a stable therapeutic effect, the administration duration of FGE was extended accordingly). Throughout this period, all groups received daily oral interventions (i.g., once daily) as follows: the Control and METH groups received an equivalent volume of saline, while the FGE, METH+FGE-L, METH+FGE-M, and METH+FGE-H groups received FGE at doses of 800, 200, 400, and 800 mg/kg (21), respectively. Body weight was monitored and recorded every 7 days.

### 3.4 Behavioral tests

Upon completion of the 28-day drug intervention, a series of behavioral tests were conducted to evaluate the effects of FGE on anxiety- and depression-like behaviors induced by METH. All tests were performed by the same experimenter to ensure consistency. To minimize olfactory cues, the testing apparatus was thoroughly cleaned with 75% ethanol after each trial. The tests proceeded as follows: the Open Field Test (OFT) was used to assess locomotor activity and anxiety-like behavior. Each animal was gently placed in the center of a square arena (45 × 45 × 45 cm) and allowed to explore freely for 10 min. The movement of each mouse was recorded and tracked using a video-tracking system. The total distance traveled and the time spent in the central area of the arena were analyzed. A longer duration spent in the center indicates lower anxiety-like behavior, while a greater total distance traveled suggests reduced depression-like behavior.

The Elevated Plus Maze (EPM) test was used to further evaluate anxiety-like behavior. The apparatus consisted of two open arms and two enclosed arms, elevated above the floor. Each animal was placed on the central platform facing an open arm and allowed to explore the maze for five minutes. The primary measure was the time spent in the open arms; a decrease in this time indicates heightened anxiety-like behavior.

The Tail Suspension Test (TST) was used to assess depression-like behavior, specifically behavioral despair. Each mouse was suspended by its tail approximately 50 cm above the table surface using adhesive tape. The test session lasted for 5 min, during which the duration of immobility was recorded. Immobility was defined as the absence of any limb or body movement, with longer immobility times indicating a greater degree of depression-like behavior.

The Forced Swim Test (FST) is a widely used paradigm for assessing depression-like behavior. Each animal was individually placed in a glass cylinder filled with water (23–25 °C) to a depth that prevented the mouse from touching the bottom. A five-minute test was conducted, with increased immobility time interpreted as enhanced despair-like behavior.

Following the completion of all behavioral tests, the mice were euthanized using a standardized method (ketamine 120 mg/kg and xylazine 8 mg/kg, intraperitoneally). Then whole brains were rapidly excised, rinsed with ice-cold saline to remove blood, gently blotted dry with filter paper, snap-frozen in liquid nitrogen, and stored at −80 °C for subsequent analysis.

### 3.5 Hematoxylin and eosin staining

Brain tissues were dehydrated through a graded ethanol series, cleared in xylene, and embedded in paraffin wax. Coronal sections of the hippocampus region were cut at 4 μm using a microtome. The sections were stained with hematoxylin and eosin (H&E) and mounted with a neutral resin. Whole-slide images of the H&E-stained sections were acquired using an EasyScan digital slide scanning system. The morphological features of the hippocampus were analyzed and photographed under a light microscope with Motic DSAssistant VM3.0 software.

### 3.6 Immunohistochemistry

Immunohistochemical staining was performed on 4-μm-thick coronal hippocampus sections to evaluate the expression of ionized calcium-binding adapter molecule 1 (Iba1), glial fibrillary acidic protein (GFAP), and neuronal nuclei (NeuN). The sections were first baked at 70 °C for one hour, then deparaffinized in xylene and rehydrated through a graded ethanol series to distilled water. Subsequently, the slides were incubated in preheated Tris/EDTA buffer (pH 9.0) and heated in a water bath at 95–100 °C for five minutes, followed by gradual cooling to room temperature.

To inhibit endogenous peroxidase activity, sections were treated with 3% hydrogen peroxide for 10 min. After washing with phosphate-buffered saline (PBS), sections were incubated for 10 min in normal goat serum blocking solution to minimize nonspecific binding. They were then incubated overnight at 4 °C with primary antibodies diluted in antibody diluent (1:200): rabbit polyclonal anti-Iba1, rabbit polyclonal anti-GFAP, and rabbit polyclonal anti-NeuN. Following thorough PBS washes to remove unbound primary antibodies, sections were incubated with a biotinylated goat anti-rabbit secondary antibody for 10 min. After another wash, sections were treated with horseradish peroxidase (HRP)-conjugated streptavidin for an additional 10 min. Immunoreactivity was visualized using a DAB substrate mixture (DAB-A: DAB-B = 1: 19) until a brown color developed. Sections were then counterstained with hematoxylin to visualize nuclei, differentiated in running tap water for 30 min, dehydrated in absolute ethanol, cleared in xylene, and finally mounted with a resinous medium. For quantitative analysis, whole-slide images were captured using a digital slide scanner. The area percentage of Iba1 and GFAP-positive staining, as well as the number of NeuN-positive cells in the hippocampus region, were quantified using image analysis software (Image-Pro Plus 6.0 or ImageJ 1.52a) by an investigator blinded to the experimental groups.

### 3.7 Quantitative real-time polymerase chain reaction

Total RNA was extracted from hippocampus tissue using TriQuick Reagent according to the manufacturer’s instructions. The concentration and purity of the extracted RNA were assessed by measuring absorbance at 260 nm and 280 nm with a spectrophotometer. Only RNA with an A260/A280 ratio between 1.8 and 2.0 was used for subsequent analysis. First-strand complementary DNA (cDNA) was synthesized from 1 μg of total RNA using the SPARKscript II RT Kit. The reverse transcription reaction was performed under the following conditions: 50 °C for 15 min, followed by enzyme inactivation at 85 °C for 5 s, and a final hold at 4 °C.

RT-PCR was performed using a SYBR Green master mix on an RT-PCR detection system. The 10 μL reaction mixture contained synthesized cDNA, specific primers, and the SYBR Green master mix. The amplification protocol consisted of an initial denaturation at 95 °C for 30 s, followed by 40 cycles of denaturation at 95 °C for 10 s, annealing at 60 °C for 10 s, and extension at 72 °C for 20 s. To confirm amplification specificity, a melt curve analysis was performed at the end of each run. The primer sequences for the target genes and the reference gene (Gapdh) are provided in Table 1.

**Table 1:** Primers sequences for real time quantitative polymerase chain reaction.

Genes	Forward primer sequence (5′-3′)	Reverse primer sequence (5′-3′)
IL-1β	AATCTCGCAGCAGCACATCA	GGAAGGTCCACGGGAAAGAC
IL-6	CTGCAAGAGACTTCCATCCAG	AGTGGTATAGACAGGTCTGTTGG
TNF-α	AAACCACCAAGTGGAGGAGC	ACAAGGTACAACCCATCGGC
IL-10	TGCAGTGTGTATTGAGTCTGCT	CGGAGAGAGGTACAAACGAGG
PI3K	AACTGACGAAGCAGGAGTGG	CCGGCTTTCTTTGTAATGGTGT
AKT	CAATGTGGGCTCATGGGTCT	GGGCCAGTTAGCATACCACA
GAPDH	CGATGCCCCCATGTTTGTGA	GAGCCCTTCCACAATGCCAA

The relative mRNA expression levels of the target genes were normalized to the Gapdh level and calculated using the comparative 2^−ΔΔCt^ method.

### 3.8 Western blot

Hippocampus tissue proteins were extracted using Radioimmunoprecipitation Assay (RIPA) lysis buffer. Equal amounts of protein samples were separated by sodium dodecyl sulfatepsolyacrylamide gel electrophoresis (SDS-PAGE) and transferred onto polyvinylidene fluoride (PVDF) membranes. The membranes were blocked with 5% skimmed milk and incubated overnight at 4 °C with primary antibodies against AKT, p-AKT, PI3K, p-PI3K, and GAPDH. After washing, the membranes were incubated for 60 min at room temperature with a horseradish peroxidase (HRP)-conjugated secondary antibody. Protein bands were visualized using an enhanced chemiluminescence (ECL) kit, and images were captured and analyzed with a gel documentation system.

### 3.9 Enzyme-linked immunosorbent assay

The levels of pro-inflammatory and anti-inflammatory cytokines, including IL-1β, IL-6, IL-10, and TNF-*α*, were quantified in hippocampus homogenates using specific commercial ELISA kits. To begin, 20 μL of the hippocampus homogenate supernatant was added to the appropriate wells of pre-coated antibody plates. After sealing the plate with a cover film, it was incubated at 37 °C for 90 min. Following incubation, the liquid was thoroughly discarded, and 100 μL of the biotinylated detection antibody working solution was added to each well. The plate was resealed and incubated for an additional hour at 37 °C. After this second incubation, the liquid was discarded again, and the wells were washed three times with 350 μL of the provided wash buffer, allowing a 1-min soak period for each wash. Next, 100 μL of horseradish peroxidase (HRP)-conjugated streptavidin working solution was added to each well. The plate was then sealed and incubated at 37 °C for 30 min. Following this incubation, the plate was washed five times as described above.

Then, 90 μL of tetramethylbenzidine (TMB) substrate solution was added to each well. The plate was resealed and incubated in the dark at 37 °C for 30 min to allow color development. The enzymatic reaction was then stopped by adding 50 μL of stop solution to each well. Finally, the optical density (OD) of each well was measured immediately at 450 nm using a microplate reader. Cytokine concentrations were determined by interpolating the OD values against the standard curve generated for each assay.

### 3.10 Statistical analysis

Quantitative data are presented as mean ± standard error of the mean (M ± SEM). Statistical analysis were performed using SPSS software (version 29.0.2.0, IBM, United States). Differences among multiple groups were assessed using one-way analysis of variance (ANOVA), followed by Bonferroni’s *post hoc* test for pairwise comparisons, and all data were tested for normality and homogeneity of variance before comparison, non-parametric tests were performed for all data that did not meet the assumptions of normality and homogeneity of variance. *p* < 0.05 was considered statistically significant.

## 4 Results

### 4.1 Target prediction via network pharmacology

**Figure 1:**
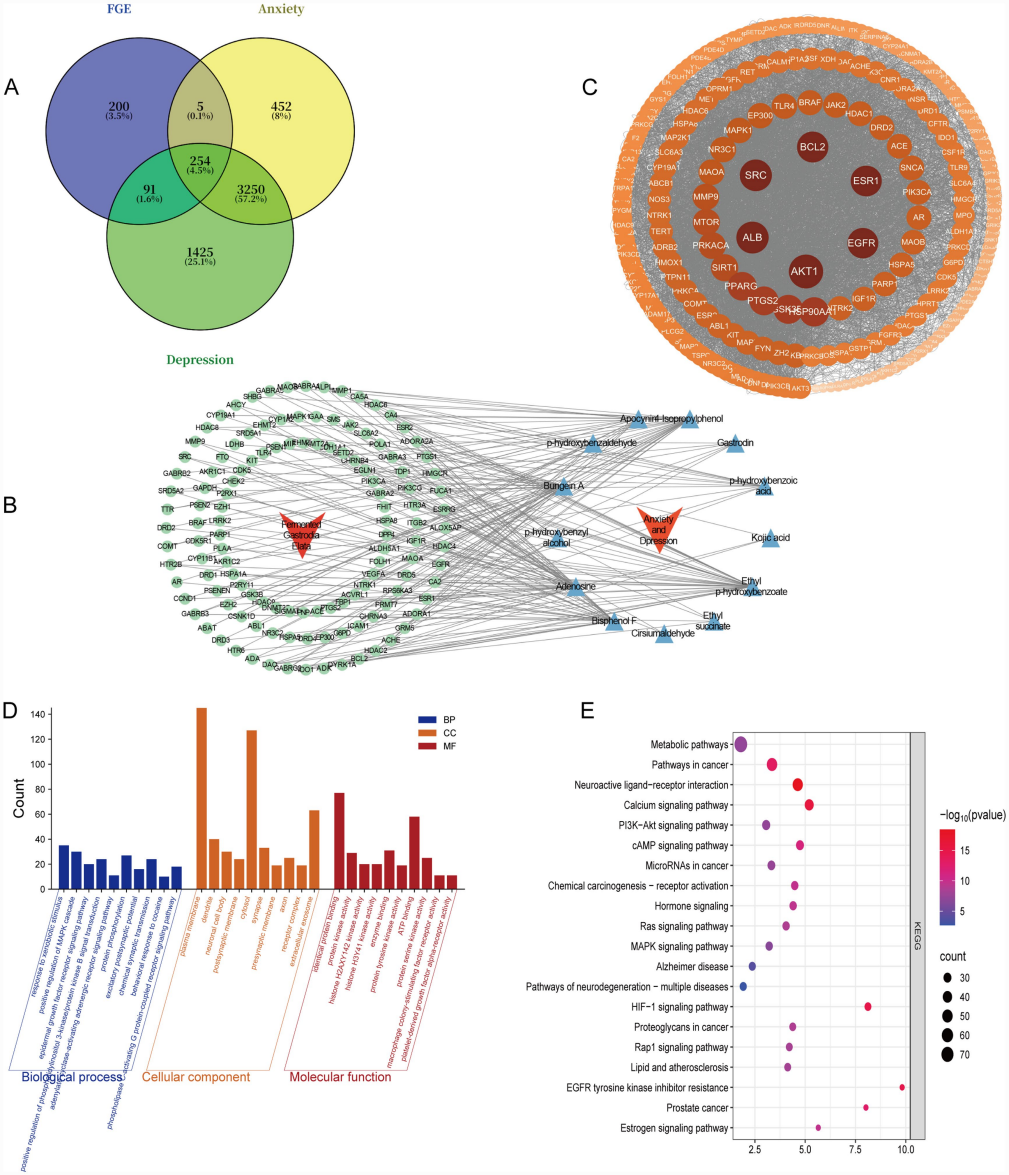
Network pharmacology predictions of FGE intervention in anxiety and depression. **(A)** Venn diagram illustrating the overlapping targets between FGE and anxiety/depression. **(B)** “Compound-Target-Disease” network of FGE visualized using Cytoscape. **(C)** Protein–protein interaction (PPI) network of core targets. Topological parameter analysis of intersecting targets identified 10 core targets meeting the criteria (degree ≥ 156, betweenness 1,387, closeness ≥0.56). Nodes represent proteins, and edges represent protein–protein associations. Lighter and larger nodes indicate higher importance. The inner ring highlights the core targets. **(D)** Circular visualization plot of the top 30 GO enrichment terms (*p* < 0.05). **(E)** Bubble plot visualization of the top 20 KEGG enrichment pathways (*p* < 0.01).

A total of 104 unique chemical components of FGE were initially identified from the PubMed database (24, 25). After applying Lipinski’s Rule of Five to evaluate drug-like properties, 13 potential active components were selected (Table 2). These components were predicted to target 550 distinct human proteins. Concurrently, 3, 961 and 5, 020 targets associated with anxiety and depression, respectively, were retrieved from relevant disease databases, including GeneCards, OMIM, and DrugBank. The intersection of these datasets revealed 254 common targets shared among the FGE components, anxiety, and depression (Figure 1A), which were considered potential core targets for the anti-anxiety and antidepressant effects of FGE. A “Compound-Target-Disease” network was constructed and visualized using Cytoscape (version 3.9.1; Figure 1B). Network analysis indicated that FGE acts through a multi-component, multi-target mechanism. The Protein–Protein Interaction (PPI) network of the 254 common targets, generated from the STRING database, identified several high-degree hub nodes (Figure 1C). The top five targets ranked by degree *p* value included SRC, ALB, AKT1, and ESR1, among others. The top 10 hub targets are detailed in Table 2. These selected gene targets are likely central to the therapeutic mechanism of FGE in addressing anxiety and depression.

**Table 2:** Thirteen active components in FGE.

No.	Components
1	Kojic acid
2	p-hydroxybenzyl alcohol
3	Apocynin
4	p-hydroxybenzoic acid
5	p-hydroxybenzaldehyde
6	Ethyl succinate
7	Ethyl p-hydroxybenzoate
8	Cirsiumaldehyde
9	4-Isopropylphenol
10	Bungein A
11	Bisphenol F
12	Gastrodin
13	Adenosine

Functional enrichment analysis of the 254 common targets was performed using the DAVID database. A total of 1, 188 GO terms were significantly enriched, including 799 terms in biological processes (BP), 114 in cellular components (CC), and 275 in molecular functions (MF). The GO analysis results suggest that the therapeutic effects of FGE on anxiety and depression may be linked to the following key processes (as shown in Figure 1D): BP: positive regulation of the MAPK cascade, excitatory postsynaptic potential, chemical synaptic transmission, and the adenylate cyclase-activating adrenergic receptor signaling pathway; CC: dendrite, neuronal cell body, and synapse; MF: protein kinase activity, protein tyrosine kinase activity, and ATP binding. KEGG pathway enrichment analysis identified the top 20 significantly enriched pathways based on enrichment scores (*p* < 0.05; see Figure 1E). The results indicate that the mechanisms of FGE primarily involve metabolic pathways, neuroactive ligand-receptor interaction, the PI3K-AKT signaling pathway, the MAPK signaling pathway, and the cAMP signaling pathway. Notably, the PI3K-AKT signaling pathway was among the most significantly enriched pathways, while the synapse emerged as a key cellular component. Based on these predictive findings, the PI3K-AKT signaling pathway and synaptic function were selected for further experimental validation.

**Figure 2:**
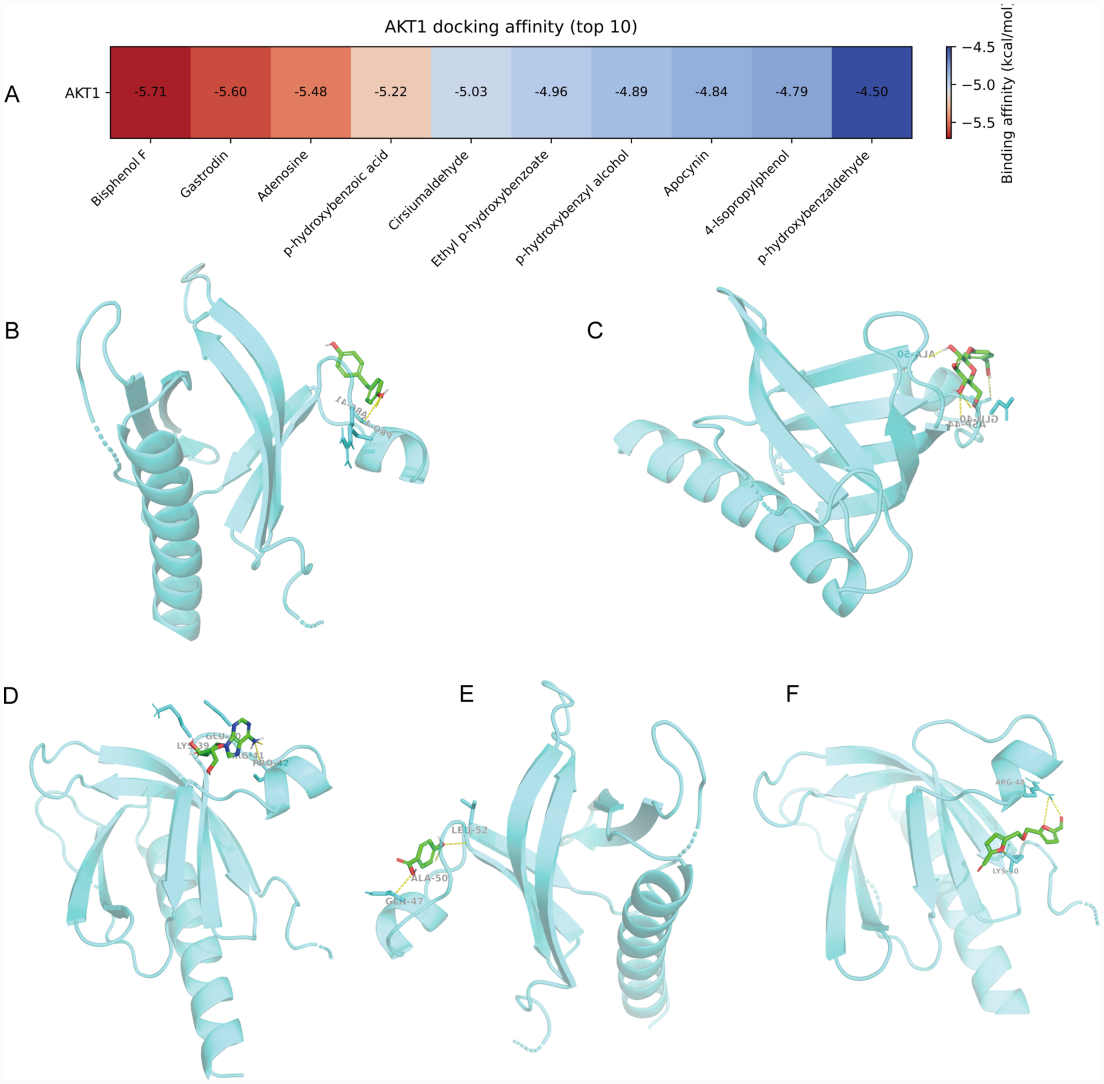
Molecular docking analysis of FGE active constituents with AKT1. **(A)** Heatmap illustrating the binding energies of 10 active constituents of FGE docked with the target AKT1. **(B–F)** Molecular docking diagrams depicting the interactions between the five key compounds and AKT1: **(B)** AKT1-Bisphenol F; **(C)** AKT1-Gastrodin; **(D)** AKT1-Adenosine; **(E)** AKT1-*p*-hydroxybenzoic acid; **(F)** AKT1-Cirsiumaldehyde.

In the preceding PPI network analysis, AKT1 emerged as the node with the highest degree, indicating its potential significance as a key target for the bioactivities of FGE. To validate this finding, molecular docking was performed to examine the interactions between AKT1 and the ten active components of FGE. The calculated binding energies ranged from −4 to −6 kcal/mol. Notably, five of these compounds exhibited binding energies below 5 kcal/mol (Figure 2A), suggesting relatively favorable binding interactions. Among them, bisphenol F demonstrated the strongest affinity for AKT1, indicating that it may be the primary bioactive compound responsible for the anxiolytic and antidepressant effects of FGE (Figures 2B–F).

### 4.2 Body weight changes in mice

**Figure 3:**
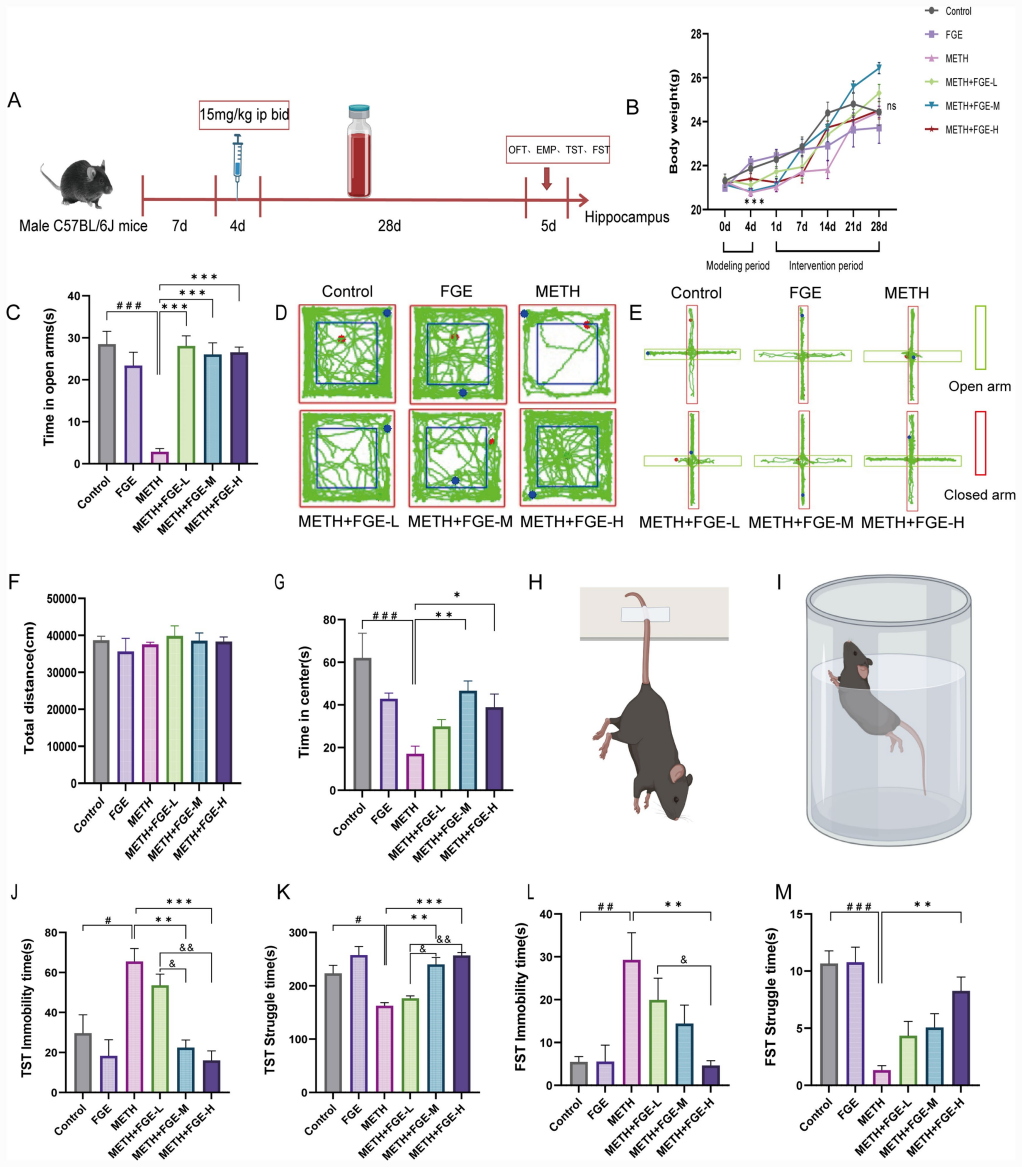
FGE ameliorates METH-induced anxiety- and depression-like behaviors in mice. **(A)** Schematic illustration of the timeline of the METH modeling and FGE intervention protocol. **(B)** Body weight changes of mice in different groups throughout the experiment. **(C)** Time spent in the open arms in the Elevated Plus Maze (EPM) test. **(D,E)** Representative movement traces of mice in the open field test (OFT) **(D)** and the Elevated Plus Maze (EPM) **(E)**. **(F,G)** Total distance traveled **(F)** and time spent in the center area **(G)** in the OFT. **(H,I)** Schematic diagrams showing the Tail Suspension Test (TST) **(H)** and the Forced Swim Test (FST) **(I)**. **(J,K)** Immobility time **(J)** and struggling time **(K)** in the TST. **(L,M)** Immobility time **(L)** and swimming time **(M)** in the FST. *p < 0.05, **p < 0.01, ***p < 0.001; #*p* < 0.05, ##*p* < 0.01, ###*p* < 0.001, &*p* < 0.05, &&*p* < 0.01, &&&*p* < 0.001. Figures 3H and 3I were created with BioRender.com.

During the METH induction phase, mice in all groups receiving METH exhibited a decline in body weight. In contrast, body weights in the Control and FGE groups increased steadily and were significantly higher than those in the METH-treated groups (*p* < 0.05). Throughout the 4-week intervention phase, body weights in all FGE intervention groups (METH+FGE-L, −M, -H) showed a stable upward trend (Figure 3A). Notably, the average body weights in these groups consistently exceeded those of the METH model group (Figure 3B). At the endpoint of the intervention, although no statistically significant difference in final body weight was observed among the groups (*p* > 0.05), the average weight of the FGE-treated groups remained higher than that of the METH group (Figure 3B). This suggests a positive trend toward weight recovery with FGE intervention.

### 4.3 FGE alleviates METH-induced anxiety- and depression-like behaviors in mice

We assessed the potential of FGE to alleviate anxiety- and depression-like behaviors in our model.

Anxiety-like Behavior: In the elevated plus maze (EPM) test, mice in the METH group spent significantly less time in the open arms compared to the control group (*p* < 0.001), indicating heightened anxiety. This METH-induced deficits was ameliorated by FGE intervention, as mice in the FGE-treated groups showed a significant increase in open-arm time relative to the METH group (Figure 3C). Similarly, in the open field test (OFT), METH-exposed mice spent considerably less time in the central area (Figures 3F,G). FGE intervention reversed these behavioral alterations, with both the medium- and high-dose FGE groups showing significant improvement (*p* < 0.05). Together, these results indicate that FGE effectively mitigates anxiety-like behaviors in METH-exposed mice (Figures 3D,E).

Depression-like behavior was further assessed using the tail suspension test (TST) and forced swim test (FST). In the TST, the duration of immobility was significantly prolonged in the METH group compared to the control group. Consistent with the anxiety test results, FGE administration reversed the METH-induced increase in immobility time. All FGE intervention groups showed a significant decrease in immobility time and a corresponding increase in struggling time compared to the METH model group (*p* < 0.05; Figures 3J,K). A similar pattern was observed in the FST, where FGE intervention effectively counteracted the METH-induced prolongation of immobility time and increased the swimming time of the mice (*p* < 0.05; Figures 3L,M). These results indicate that FGE also alleviates depression-like behaviors in mice challenged with METH.

### 4.4 FGE attenuates METH-induced pathological damage in the hippocampus

**Figure 4:**
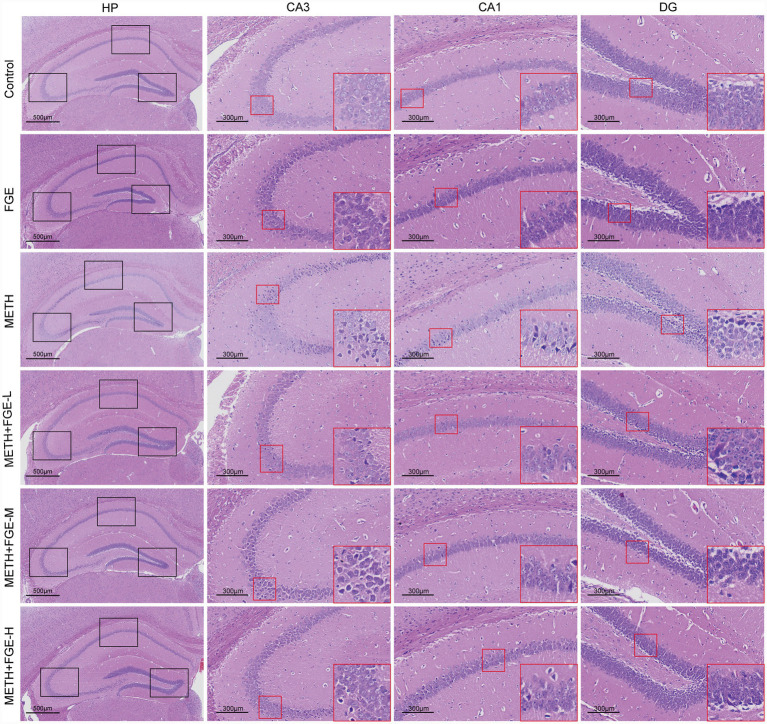
FGE attenuates METH-induced histopathological damage in the hippocampus (H&E). Red arrows indicate necrotic neurons with eosinophilic changes.

Histopathological changes in the hippocampus tissue were assessed using H&E staining (Figure 4). In the Control group, hippocampus neurons in the CA1 and CA3 regions exhibited regular morphology, an orderly arrangement, and clear nuclear boundaries. In stark contrast, the METH group displayed significant pathological alterations. Granular and pyramidal cells in the CA1 and CA3 regions showed a disordered arrangement, with evidence of scattered eosinophilic staining changes suggestive of neuronal damage. Notably, intervention with FGE markedly attenuated these METH-induced histopathological changes. Hippocampus neurons in the FGE-intervened groups demonstrated a more organized arrangement and improved cellular density compared to the METH group, indicating a protective effect of FGE against neuronal injury.

### 4.5 FGE suppresses microglial and astrocytic activation and neuroinflammation in METH-treated mice

**Figure 5:**
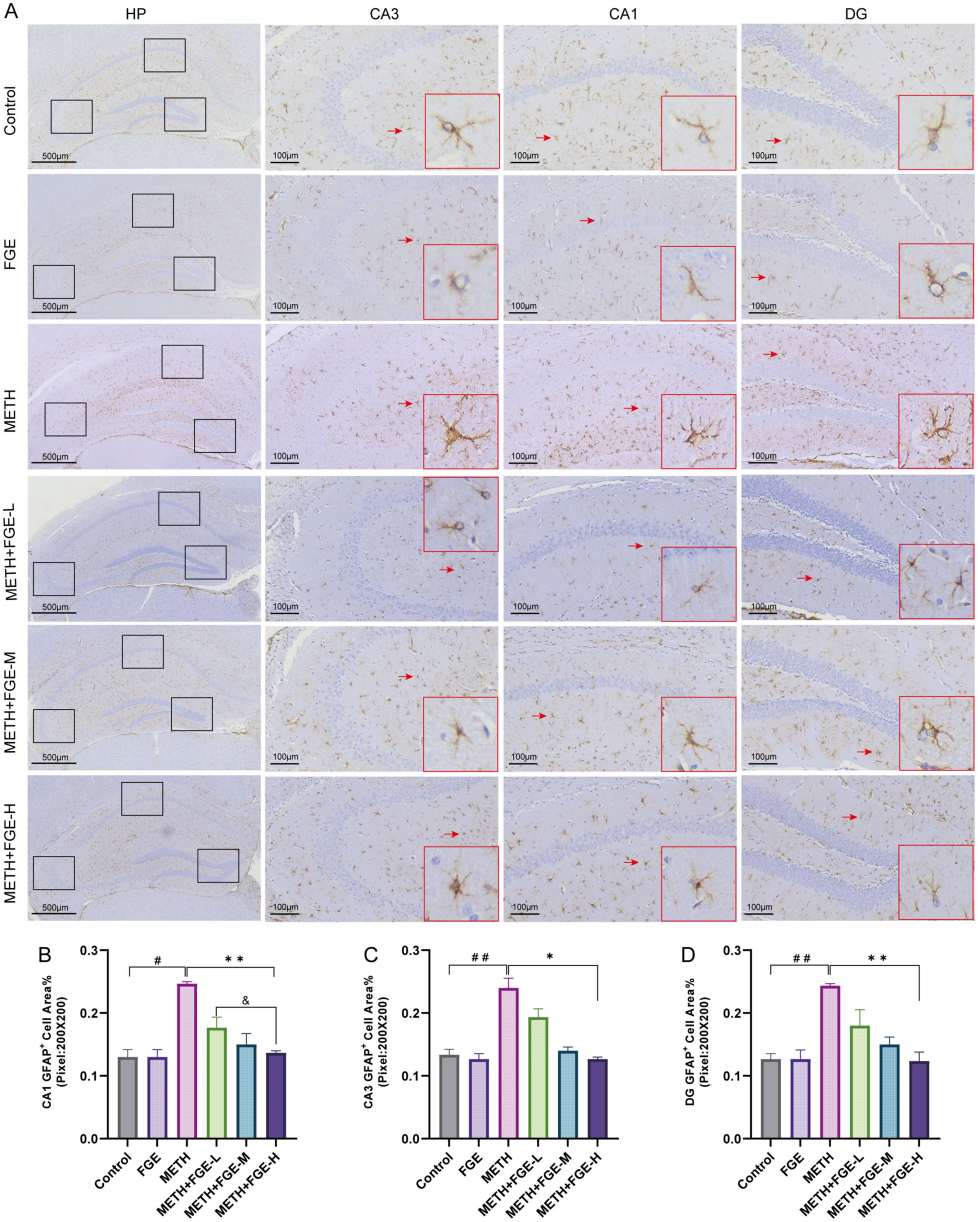
FGE suppresses METH-induced astrocyte activation in the mouse hippocampus. **(A)** Representative immunohistochemical images showing GFAP expression (an astrocyte marker) in the hippocampus across different groups. Red arrows indicate activated astrocytes exhibiting a hypertrophic morphology. **(B–D)** Quantitative analysis of the GFAP-positive area percentage. *p < 0.05, **p < 0.01, ***p < 0.001; #*p* < 0.05, ##*p* < 0.01, ###*p* < 0.001, &*p* < 0.05, &&*p* < 0.01, &&&*p* < 0.001.

**Figure 6:**
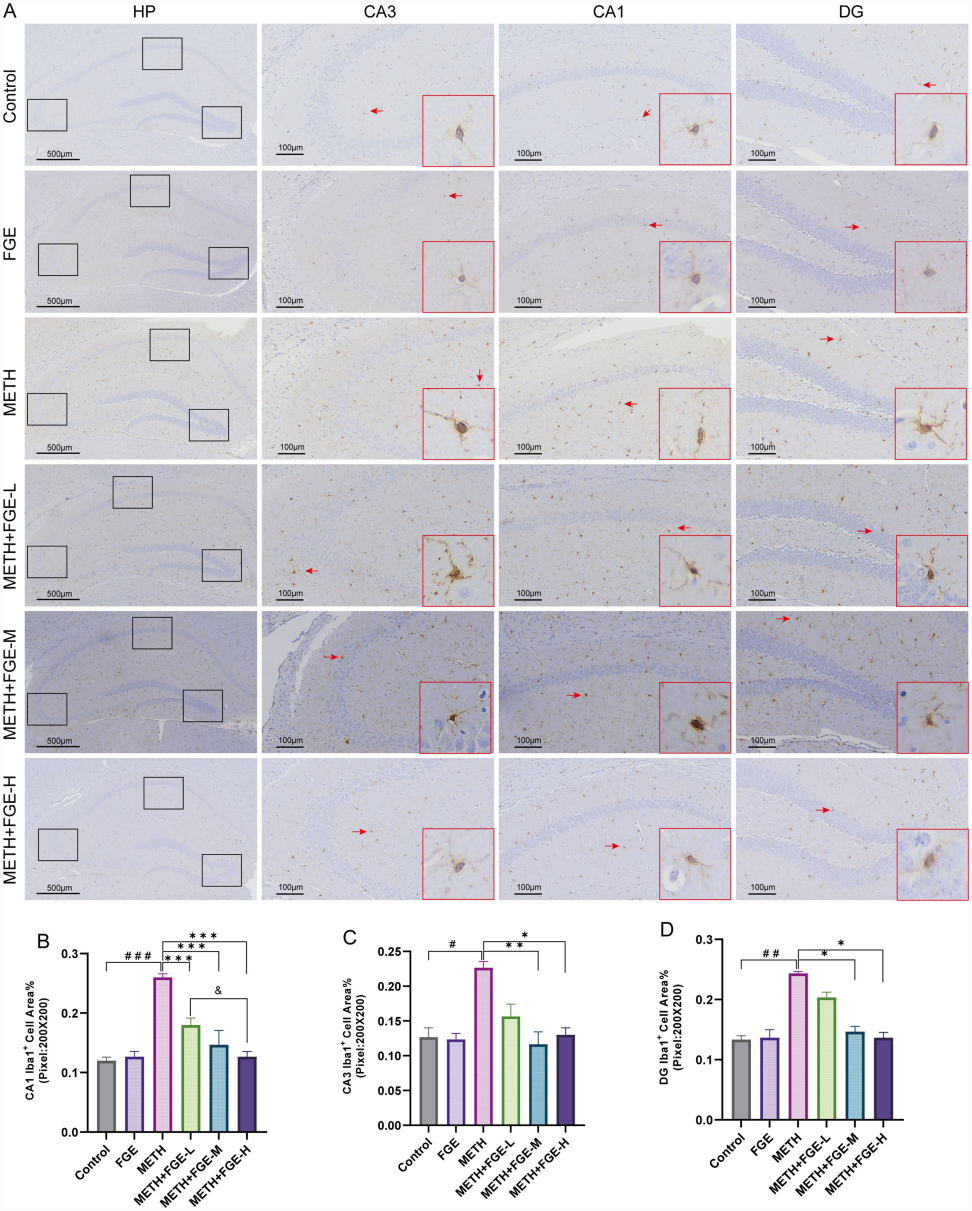
FGE inhibits METH-induced microglial activation in the mouse hippocampus. **(A)** Representative immunohistochemical images of Iba1 expression in the hippocampus. Red arrows indicate activated microglia with an amoeboid morphology. **(B–D)** Quantitative analysis of the percentage of Iba1-positive area. *p < 0.05, **p < 0.01, ***p < 0.001; #*p* < 0.05, ##*p* < 0.01, ###*p* < 0.001, &*p* < 0.05, &&*p* < 0.01, &&&*p* < 0.001.

The activation of microglia and astrocytes, a key process in neuroinflammation progression, was evaluated through IHC staining for their respective markers, Iba-1 and GFAP (26, 27). As shown in Figures 5, 6, METH exposure significantly increased GFAP and Iba-1 positive staining compared to the Control group (*p* < 0.05), indicating pronounced glial cell activation. Morphologically, astrocytes in the METH group shifted from a typical star-like shape to a more rounded or hypertrophic form, characterized by shortened processes and increased branching (Figure 5). Similarly, microglia transitioned from a ramified, resting state to an activated state, displaying elongated processes, increased branching, larger cell body area, and a typical amoeboid morphology (Figure 6). These observations align with previously reported features of glial activation (27–29). Intervention with FGE substantially attenuated the METH-induced changes. Compared to the METH group, FGE treatment ameliorated the morphological alterations in both microglia and astrocytes, resulting in reduced activation levels, as evidenced by decreased Iba1 and GFAP immunoreactivity.

**Figure 7:**
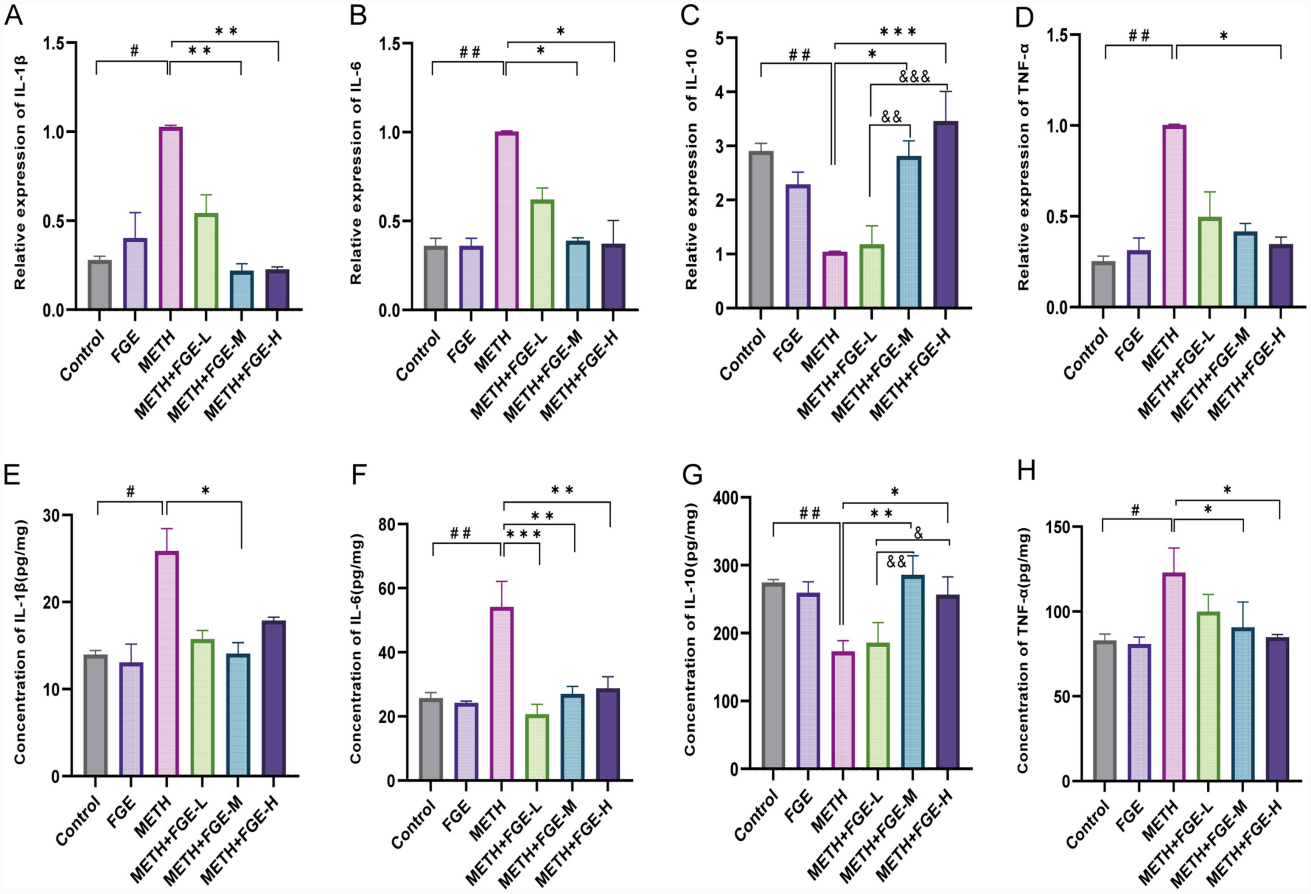
FGE modulates the expression of inflammatory cytokines in the hippocampus of METH-exposed mice. mRNA expression levels of IL-1β **(A)**, IL-6 **(B)**, IL-10 **(C)**, and TNF-*α* **(D)** in hippocampus tissues, as determined by RT-qPCR. Gene expression was normalized to Gapdh and is presented as the fold change relative to the METH group. **(E–H)** Protein levels of IL-1β, IL-6, IL-10, and TNF-α in hippocampus homogenates, as measured by ELISA. *p < 0.05, **p < 0.01, ***p < 0.001; #*p* < 0.05, ##*p* < 0.01, ###*p* < 0.001, &*p* < 0.05, &&*p* < 0.01, &&&*p* < 0.001.

Having established that FGE alleviates METH-induced morphological activation of microglia and astrocytes, we next investigated whether it could reverse the associated functional changes, specifically the secretion of inflammatory mediators. Activated glial cells are a primary source of pro-inflammatory cytokines, making this an important area of study (30, 31). qPCR analysis revealed that, compared to the Control group, METH exposure significantly upregulated the mRNA expression of pro-inflammatory cytokines in the hippocampus, including IL-1β, IL-6, and TNF-*α*, with an approximate 3- to 4-fold increase (*p* < 0.05; Figures 7A,B,D). Intervention with FGE significantly attenuated this upregulation of pro-inflammatory factors while simultaneously enhancing the expression of the anti-inflammatory cytokine IL-10 (*p* < 0.05; Figure 7C). This gene expression analysis was further supported by ELISA, which confirmed a consistent pattern of cytokine changes at the protein level (Figures 7E,G,H). These findings suggest that FGE can counteract the METH-induced hyperactivation of microglia and astrocytes, shifting their functional state from pro-inflammatory to anti-inflammatory by decreasing pro-inflammatory cytokine levels and increasing anti-inflammatory cytokine expression.

**Figure 8:**
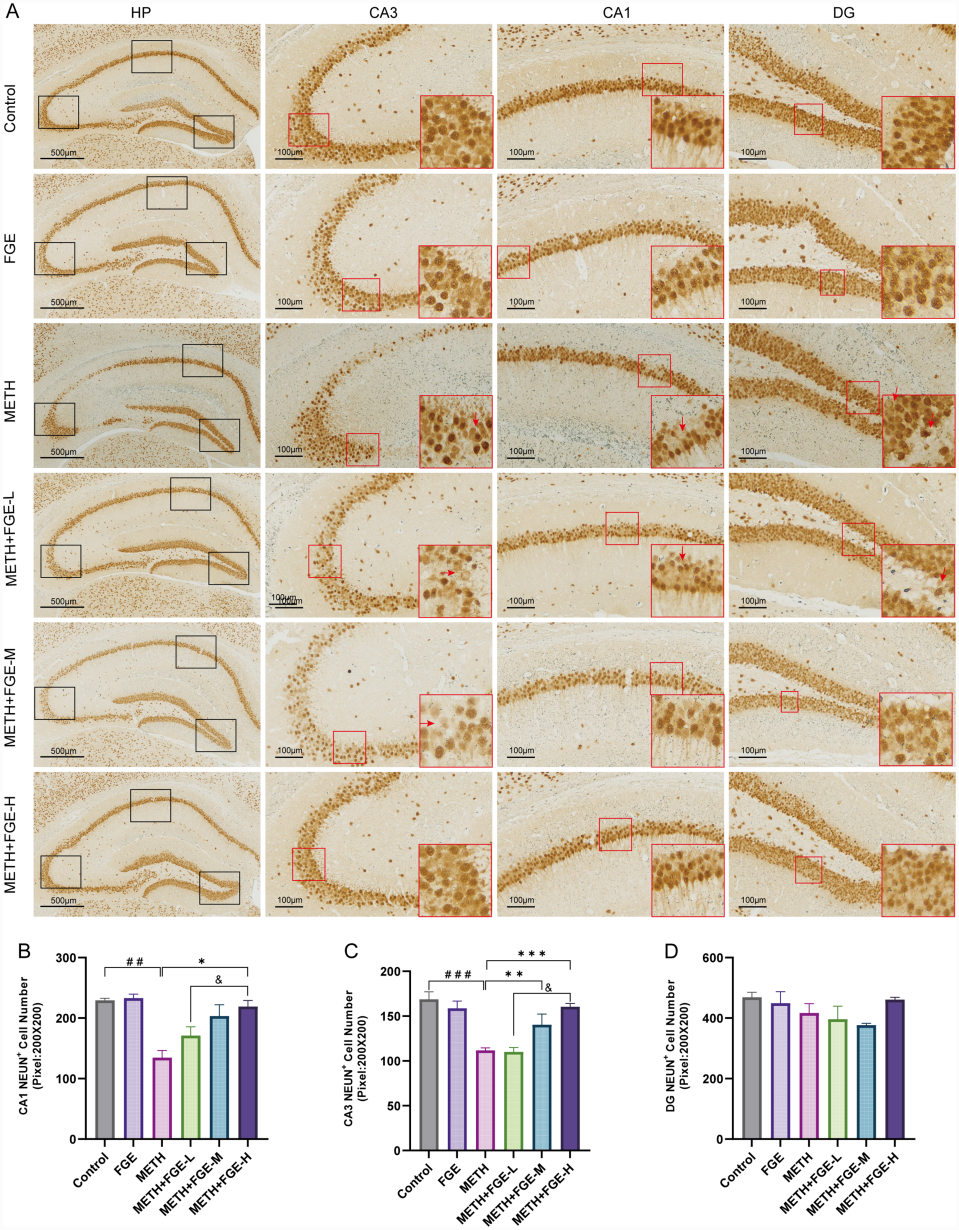
FGE mitigates METH-induced neuronal loss in the mouse hippocampus. **(A)** Representative immunohistochemical images of NeuN expression in the hippocampus CA1, CA3, and DG regions across different groups. Red arrows indicate examples of pyknotic or damaged neurons. **(B–D)** Quantitative analysis of the number of NeuN-positive cells in the hippocampus CA1, CA3, and DG regions. *p < 0.05, **p < 0.01, ***p < 0.001; #*p* < 0.05, ##*p* < 0.01, ###*p* < 0.001, &*p* < 0.05, &&*p* < 0.01, &&&*p* < 0.001.

Activated immune cells, including microglia and astrocytes, release neuroinflammatory factors that contribute to neuronal injury. These factors disrupt neuronal membranes, impair ionic balance, interfere with neurotransmitter transmission, and ultimately trigger neuronal apoptosis (32). Building on the observed neuroinflammatory response, we next evaluated neuronal damage in the hippocampus CA1, CA3, and dentate gyrus (DG) regions using NeuN immunohistochemistry. As illustrated in Figure 8, the METH group exhibited a significant reduction in the number of NeuN-positive cells in both the CA1 and CA3 regions compared to the Control group (*p* < 0.01). Additionally, the remaining neurons displayed a disordered arrangement, with evidence of scattered pyknotic nuclei and neuronal loss, indicating that METH severely disrupts neuronal structure and function. Notably, treatment with FGE significantly mitigated this neuronal damage. The FGE-treated groups, especially the high-dose group (*p* < 0.05), demonstrated a marked increase in the number of NeuN-positive cells and a more organized cellular arrangement in the CA1 and CA3 regions compared to the METH group. In contrast, no significant differences in the number or morphology of NeuN-positive cells were observed in the dentate gyrus (DG) across all groups.

### 4.6 Effect of FGE on the expression of PI3K-AKT pathway-related genes and proteins in the METH group

**Figure 9:**
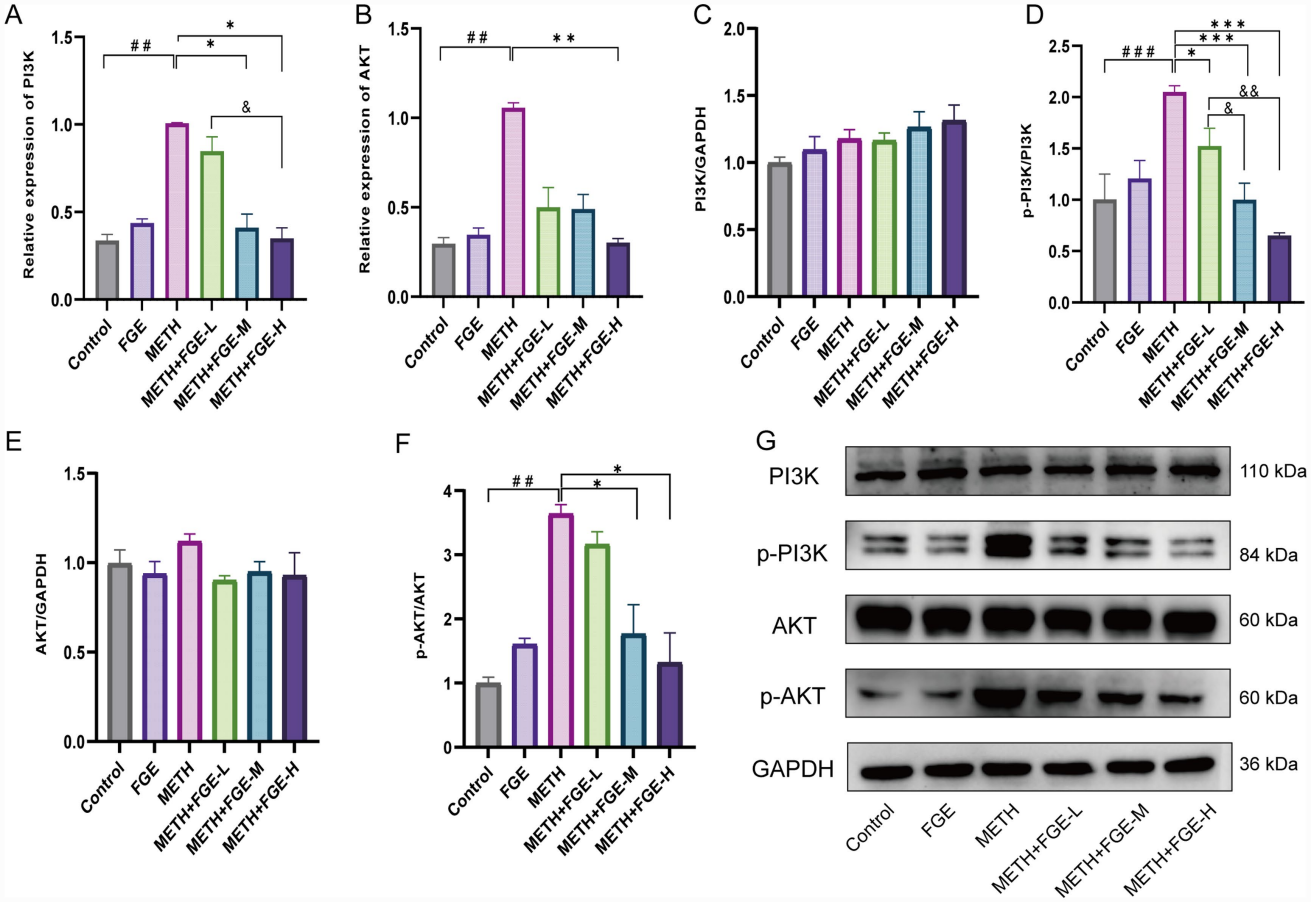
Anti-anxiety and antidepressant effects of FGE on METH-treated mice through regulation of PI3K/AKT signaling pathway-related gene and protein expression. **(A,B)** Representative qPCR results of PI3K/AKT pathway genes and proteins in the hippocampus across all mouse groups. **(C,E)** Levels of PI3K and AKT proteins in the hippocampus of METH-treated mice. **(D,F)** Levels of p-PI3K and p-AKT proteins in the hippocampus of METH-challenged mice. **(G)** Representative Western blot images showing protein levels of PI3K, p-PI3K, AKT, p-AKT, and GAPDH in different groups. *p < 0.05, **p < 0.01, ***p < 0.001; #*p* < 0.05, ##*p* < 0.01, ###*p* < 0.001, &*p* < 0.05, &&*p* < 0.01, &&&*p* < 0.001.

Quantitative PCR (qPCR) analysis revealed significantly higher mRNA levels of PI3K and AKT in the METH group compared to the Control group (*p* < 0.05). Notably, FGE intervention effectively normalized the expression of these genes (Figures 9A,B). Western blot analysis was performed to evaluate the expression of proteins associated with the PI3K-AKT pathway in the hippocampus across the experimental groups. As shown in Figure 9, no significant differences were observed in total PI3K and AKT protein levels among the groups (Figures 9C,E,G). However, METH stimulation significantly increased the relative expression ratios of p-PI3K/PI3K and p-AKT/AKT in hippocampus tissues (Figures 9D,F). In contrast, treatment with FGE at doses of 400 and 800 mg/kg significantly reduced the phosphorylation levels of both PI3K and AKT. These findings suggest that inhibition of the PI3K/AKT signaling pathway may be a key mechanism underlying the antidepressant-like effects of FGE.

## 5 Discussion

In our study, network pharmacology analysis indicated that the potential targets of FGE’s active components, along with established targets linked to anxiety and depression, are significantly enriched in the PI3K-AKT signaling pathway. This computational prediction was confirmed by qPCR and Western blot results, which demonstrated a significant upregulation of PI3K and AKT gene and protein expression in the hippocampus of METH-exposed mice. This convergence of evidence suggests that the neuroprotective effects of FGE against METH-induced neurotoxicity are likely mediated, at least in part, through the modulation of the PI3K-AKT signaling pathway. Collectively, our findings highlight a novel research direction for using natural fermented products, such as FGE, to address METH-related emotional disorders. The hippocampus, a key structure of the limbic system located between the thalamus and the temporal lobe, plays a crucial roles in regulating behavior, emotion, and cognition (27).

Our study demonstrated that METH exposure resulted in significant weight loss and induced anxiety- and depression-like behaviors. It also caused neuronal disorganization and apoptosis in the hippocampus CA1 and CA3 regions (Figures 4, 8). Notably, we observed no significant morphological changes or neuronal loss in the DG. This region-specific vulnerability aligns with previous reports indicating that METH-induced neuronal damage predominantly affects the CA1 region while largely sparing the DG (33, 34). This differential susceptibility is often attributed to the inherent capacity for neurogenesis and ongoing renewal of granule cells within the DG. Following FGE intervention, the final body weight of the FGE-treated mice did not differ significantly from that of the Control group, though both groups consistently weighed more than the METH group throughout the study. This may reflect the natural recovery capacity of the mice, which was potentially enhanced by the FGE intervention. Notably, FGE intervention significantly alleviated the anxiety- and depression-like behaviors induced by METH.

Further investigation revealed that METH exposure induced significant hyperactivation of microglia and astrocytes in the mouse hippocampus, characterized by glial proliferation, somatic hypertrophy, increased process branching, and abnormal secretion of inflammatory cytokines (Figures 5, 6). Pathological overactivation of microglia and astrocytes is a hallmark of various psychiatric disorders, including depression and anxiety (29). The underlying mechanism primarily involves the excessive release of inflammatory factors by these glial cells, which disrupts hippocampus neuro plasticity and impairs synaptic signaling (29, 30, 33, 34). Our results demonstrate that FGE intervention effectively suppressed METH-induced activation of both microglia and astrocytes in the hippocampus. This suppression was evidenced by reduced pro-inflammatory cytokine levels and increased anti-inflammatory cytokine levels. Additionally, FGE intervention ameliorated neuronal damage in the CA1 and CA3 regions. Notably, these protective effects showed a positive correlation with the dosage of FGE administered. Collectively, these findings suggest that the therapeutic benefits of FGE are likely dose-dependent. The underlying mechanism appears to involve the attenuation of neuroinflammation, which subsequently protects against neuronal injury and reverses the anxiety- and depression-like behaviors observed in METH-exposed mice.

Previous studies have demonstrated the effectiveness of Gastrodia elata in alleviating various central nervous system disorders. The primary bioactive constituents of this plant include GABA, gastrodin, 4-hydroxybenzyl alcohol (4-HBA), and 4-hydroxybenzaldehyde (PHBA). Compared to the raw Gastrodia elata material, FGE likely exhibits enhanced potency, which may be attributed to the increased bioavailability of its active components or the production of new bioactive metabolites during fermentation (19, 35). The neuroprotective effects of FGE are primarily mediated through multiple mechanisms, including scavenging reactive oxygen species (ROS), inhibiting the release of pro-inflammatory cytokines, and potentiating GABAergic neurotransmission, ultimately mitigating oxidative stress and neuronal apoptosis (36, 37). In contrast to conventional anxiolytic and antidepressant drugs, FGE contains a diverse array of active compounds such as phenolic compounds and their glycosides, polysaccharides, sterols, and organic acids (35). This compositional complexity provides a unique advantage by allowing therapeutic effects to be exerted through multiple targets and pathways. For instance, 4-HBA and PHBA can modulate serotonergic and GABAergic systems, which in turn confers anti-inflammatory, sedative-hypnotic, and anxiolytic effects (25, 38). They also significantly inhibit oxidative stress and excitotoxicity, reducing neuronal death in the hippocampus CA1 region (39); Additionally, Gastrodia elata polysaccharides have been shown to ameliorate cognitive function in APP/PS1 transgenic mice by facilitating the clearance of amyloid-*β* (Aβ) plaques and increasing neuronal survival (40). Furthermore, studies have indicated that the fermentation process significantly increases the content of key active ingredients in FGE, such as 4-HBA and parishin B (25). The elevated concentration of these bioactive compounds is likely a crucial factor contributing to the superior anti-anxiety and antidepressant efficacy of FGE compared to its non-fermented counterpart.

This study provides the first evidence supporting the therapeutic potential of FGE against METH-induced neurotoxicity and behavioral deficits. Our findings were initially guided by a network pharmacology approach; however, several inherent limitations in the study of complex natural products must be acknowledged. The primary limitation stems from the multi-component, multi-target, and multi-pathway nature of FGE. Although network pharmacology offers a robust predictive framework, this study did not precisely identify the specific active constituents within FGE responsible for the observed effects, nor did it determine which components can effectively cross the blood–brain barrier to act in the central nervous system. For example, studies have shown that gastrodin can cross the blood–brain barrier and is metabolized to produce 4-HBA, both of which contribute to the pharmacological effects (41, 42). While GABA is a small molecule (molecular weight 103 g/mol), its high water solubility and polar nature result in extremely low efficiency in crossing the blood–brain barrier (43). Therefore, a clear analysis of the drug components in FGE that can actually penetrate the blood–brain barrier and enter the brain would make the conclusions of this study more convincing. This limitation represents a shortcoming of the current research, and we will subsequently incorporate this work into the overall research framework for improvement. Furthermore, the exact molecular mechanisms underlying the modulation of the PI3K-AKT signaling pathway and glial cell activation have not been fully elucidated.

Future research should employ advanced integrated omics technologies, such as proteomics and metabolomics, to systematically identify the key bioactive components of FGE and their corresponding targets in the brain. Subsequent *in vitro* studies using specific receptor antagonists or gene silencing techniques are warranted to validate the precise molecular pathways involved. Ultimately, well-designed clinical trials will be essential to assess the translational potential and practical application of FGE in treating substance use disorder-related psychiatric symptoms.

## 6 Conclusion

In conclusion, the results of this study demonstrate that after intervention with FGE, METH-induced anxiety- and depression-like behaviors in mice were alleviated, which may be attributed to the pharmacological activity of FGE. According to network pharmacology prediction analysis, the underlying mechanism may be associated with the modulation of the PI3K-AKT signaling pathway. This pathway may suppress the hyperactivation of microglia and astrocytes, reduce neuroinflammation, and protect hippocampal neurons from damage. These findings provide key signaling pathways and molecular mechanisms for subsequent studies aiming to demonstrate that FGE can alleviate METH-induced emotional disorders.

## Data Availability

The original contributions presented in the study are included in the article/supplementary material, further inquiries can be directed to the corresponding authors.

## Footnotes

1
https://news.un.org/zh/story/2024/06/1129551

2
http://herb.ac.cn/

3
https://www.swisstargetprediction.ch/

4
https://ngdc.cncb.ac.cn/databasecommons/database/id/723

5
https://www.omim.org/

6
https://ngdc.cncb.ac.cn/databasecommons/database/id/296

7
https://cn.string-db.org/

8
https://davidbioinformatics.nih.gov/
